# Assessment of Psychotic Symptoms in Individuals Exposed to Very High or Extreme Altitude: A Field Study

**DOI:** 10.1089/ham.2020.0210

**Published:** 2021-12-13

**Authors:** Katharina Hüfner, Fabio Caramazza, Agnieszka E. Stawinoga, Evelyn R. Pircher Nöckler, Paolo Fusar-Poli, Sanjeeb S. Bhandari, Buddha Basnyat, Monika Brodmann Maeder, Giacomo Strapazzon, Iztok Tomazin, Barbara Sperner-Unterweger, Hermann Brugger

**Affiliations:** ^1^ University Hospital of Psychiatry II, Department of Psychiatry, Psychotherapy and Psychosomatics, Innsbruck Medical University, Innsbruck, Austria.; ^2^ Institute of Mountain Emergency Medicine, Eurac Research, Bolzano, Italy.; ^3^ Management and Committees, Eurac Research, Bolzano, Italy.; ^4^ Department of Psychosis Studies, Institute of Psychiatry, Psychology and Neuroscience, King's College, London, United Kingdom.; ^5^ Department of Brain and Behavioral Sciences, University of Pavia, Pavia, Italy.; ^6^ Mountain Medicine Society of Nepal, Kathmandu, Nepal.; ^7^ Oxford University Clinical Research Unit, Patan Academy of Health Science, Nepal International, Kathmandu, Nepal.; ^8^ Department of Emergency Medicine, University Hospital Bern and Bern University, Bern, Switzerland.; ^9^ Department of Family Medicine, Faculty of Medicine, University of Ljubljana, Ljubljana, Slovenia.; ^10^ Mountain Rescue Association of Slovenia, Kranj, Slovenia.

**Keywords:** HAPSY-Q, high altitude, high altitude cerebral edema, M.I.N.I Interview, PQ-16, psychosis

## Abstract

Hüfner, Katharina, Fabio Caramazza, Agnieszka E. Stawinoga, Evelyn R. Pircher Nöckler, Paolo Fusar-Poli, Sanjeeb S. Bhandari, Buddha Basnyat, Monika Brodmann Maeder, Giacomo Strapazzon, Iztok Tomazin, Barbara Sperner-Unterweger, and Hermann Brugger. Assessment of psychotic symptoms in individuals exposed to very high or extreme altitude: A field study. *High Alt Med Biol.* 22:369–378, 2021.

***Background:*** Symptoms of psychosis such as hallucinations can occur at high or extreme altitude and have been linked to accidents on the mountain. No data are available on how to assess such symptoms in the field and what their prevalence or predisposing factors might be.

***Methods:*** In this field study at Everest Base Camp (5,365 m) in Nepal, 99 participants of organized expeditions underwent 279 assessments: The High Altitude Psychosis Questionnaire (HAPSY-Q), the Prodromal Questionnaire, 16-items (PQ-16), and the Mini International Neuropsychiatric Interview (M.I.N.I., psychosis section) were collected together with further clinical data. Statistical analysis was done for each phase, that is, altitude range of the climb, and overall data.

***Results:*** One of 97 climbers fulfilled the M.I.N.I. diagnostic criteria for psychosis during one acclimatization climb. At least one endorsed item on the HAPSY-Q and the PQ-16, indicating the presence of symptoms of psychosis in the absence of a psychotic disorders, were identified in 10/97 (10.3%) and 18/87 (20.7%) participants respectively. The scores of the HAPSY-Q and the PQ-16 were correlated (*r* = 0.268, *p* < 0.001). Odds ratio analysis identified an increased risk for accidents in individuals with endorsed items on the HAPSY-Q.

***Conclusions:*** The diagnosis of high altitude psychosis is rare in climbers during organized expeditions. Nevertheless, subdiagnostic symptoms of psychosis occurred in a significant proportion of climbers. Future research is needed to validate these pilot findings.

## Background

Medical knowledge on somatic symptoms, which can occur at high altitude (HA), has improved over the past decades, as has the awareness of such symptoms in mountain climbers and mountaineers. Aside from somatic symptoms, also psychiatric symptoms can occur at HA. While somatic HA-related symptoms such as acute mountain sickness (AMS) have received substantial attention in medical literature, not much is known about the occurrence of psychiatric symptoms at HA. An association of HA psychosis with accidents on the mountain has been described recently, highlighting the need for further investigations into psychiatric symptoms at HA (Hufner et al., 2018). The *Diagnostic and Statistical Manual of Mental Disorders*, fifth edition (DSM-5), lists the following core symptoms for the dimensional assessment of psychosis: hallucinations, delusions, disorganized speech, abnormal psychomotor behavior, negative symptoms; and additionally impaired cognition, depression, and mania (American Psychiatric Association, 2013). Two of these symptoms have to be present for the diagnosis of psychosis, with one of them being hallucinations, delusions, or disorganized speech. Symptoms of perceptual instability, that is, hallucinations and delusions, are the defining and prevalent feature of psychosis (Horga and Abi-Dargham, 2019). Psychotic symptoms are transdiagnostic (Fusar-Poli et al., 2017); they are hallmark of schizophrenia, but can also occur in mood or substance abuse disorders, or as part of the syndrome of organic brain dysfunction (classified as delirium in DSM-5) (American Psychiatric Association, 2013).

In the recently identified entity of isolated HA psychosis, these symptoms occurred independent of organic symptoms and were found to be quickly reversible once lower altitudes were reached (Hufner et al., 2018). Most climbers who suffered from isolated HA psychosis were able to descend from the mountain on their own (unless the psychotic symptoms led them into dangerous situations), which is generally not the case in patients with high altitude cerebral edema (HACE) (Hufner et al., 2018). However, apart from the entity of isolated HA psychosis, symptoms of psychosis on the mountain can also occur as part of the syndrome of organic brain dysfunction (“delirium”). Delirium at HA can occur in the context of HACE or as a result of systemic conditions such as infection or dehydration (Ryn, 1988; Brugger et al., 1999; Garrido et al., 2000; Basnyat, 2002). Hypoxia *per se* has a pro-hallucinogenic effect (Lempert et al., 1994). Extreme environmental conditions in conjunction with sensory and social deprivation, exhaustion, psychological stress, and lack of sleep can also predispose toward psychotic symptoms (Daniel and Mason, 2015; Meyhofer et al., 2017; Carbone et al., 2020). Dysfunction of the temporoparietal cortex has been suggested to explain the unexpected and rapid onset of symptoms of isolated HA psychosis in the absence of HACE (Firth and Bolay, 2004). Dysfunction of neuronal discharges may also be reflected by seizures occurring at altitude (Hennis et al., 2014).

In recent years, a whole branch of psychiatry has committed to identifying individuals with prodromal symptoms of psychosis to deliver tailored treatment for individuals at risk of developing full-blown psychosis. The ultra high risk (UHR) syndrome is a state when subthreshold psychotic experiences emerge and during which the risk of developing psychosis is far higher than in the general population (Fusar-Poli et al., 2013). Researchers and clinicians working on UHR have developed several instruments sensitive of detecting single and early symptoms of psychosis such as the Comprehensive Assessment of At-Risk Mental State (CAARMS) Interview (Yung et al., 2005) or the self-rating Prodromal Questionnaire, 16-items (PQ-16), questionnaire (Ising et al., 2012).

The primary aim of this field study on HA psychosis was to evaluate psychotic symptoms (independent of the underlying condition) in climbers on the mountain using the newly developed questionnaire for psychotic symptoms at HA (HAPSY-Q), a screening tool for prodromal symptoms of psychosis (PQ-16), and a physician-based rating of psychosis (Mini International Neuropsychiatric Interview [M.I.N.I.], psychosis module). In addition, data on possible risk factors such as infection or AMS and protective factors were collected. Since psychosis has been shown to be associated with accidents on the mountain previously (Hufner et al., 2018), we hope that our research will help to reduce accidents and near-accidents on the mountain.

## Methods

### Study setting

**FIG. 1.**
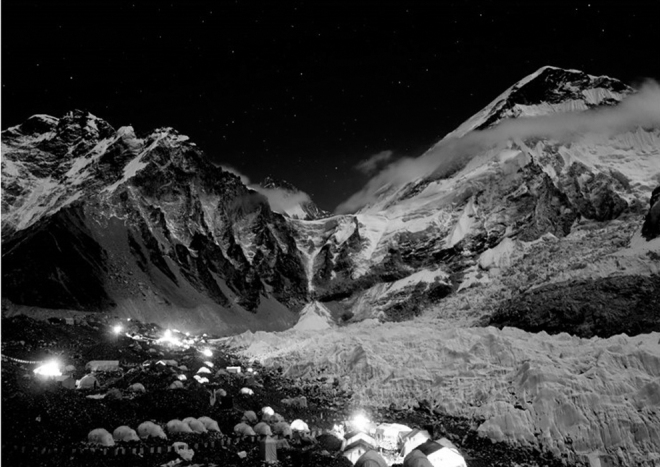
Study site of the field study, EBC (© Fabio Caramazza). EBC, Everest Base Camp.

This is a field study with a longitudinal design. Data were collected by author F.C. during April and May 2019 at Everest Base Camp (EBC) (5,365 m asl), Khumbu region, Nepal (Fig. 1). Questionnaires were completed digitally on a tablet PC. Twelve questionnaires collected at EBC by author S.S.B. in a pilot study in 2018 were also included. The study was approved by Nepal Health Research Council. Informed written consent was obtained from all participants before inclusion in the study.

### Questionnaires and clinical examination

An interdisciplinary international team established the questions and selected the standardized questionnaires to be used in this field study. The study was designed along the lines of the STAR Data Reporting Guidelines for Clinical High Altitude Research (Brodmann Maeder et al., 2018).

The following data were collected as self-ratings: sociodemographic data, data on lifestyle habits and past medical history, data related to the altitude climb, including data on accidents or near accidents, the High Altitude Psychosis Questionnaire (HAPSY-Q) (Hüfner et al., 2019), the PQ-16 (Ising et al., 2012), and the Lake Louise AMS score (Roach et al., 2018). The second part consisted of observer-based ratings, including the Confusion Assessment Method (CAM) for the detection of delirium (Inouye et al., 1990), the Mini International Neuropsychiatric Interview English Version 7.0.1 for DSM-5 (M.I.N.I. psychosis module) (Sheehan et al., 1998; American Psychiatric Association, 2013), and the evaluation for the presence of signs and symptoms of high altitude pulmonary edema (HAPE) or HACE. For the latter two conditions, the HAPE and HACE scoring algorithm deducted from the STAR Data reporting guidelines was used. All the participants underwent a brief clinical examination, including a basic neurological examination with emphasis on signs of ataxia and changes in mental status, and a chest auscultation. The diagnosis of HACE was made in persons with AMS+ataxia AND/OR mental status change or in individuals without AMS with ataxia+mental status change. The diagnosis of HAPE was diagnosed by assessing the presence of at least two main criteria (weakness/decreased exercise performance, dyspnea at rest, cough, or chest tightness or congestion) and at least two supplemental criteria (tachypnea, tachycardia, rales or wheezing in at least one lung field, or central cyanosis) (Brodmann Maeder et al., 2018; Roach et al., 1993).

Pre-existing cutoff values for the PQ-16 and the HAPSY-Q were assessed in the literature. The cutoff for the PQ-16 to identify “at-risk” cases was defined as ≥6 by Ising et al. (2012) with a sensitivity and specificity of 87% and McDonald et al. (2019), and as ≥5 according to Savill et al. (2018). The defined cutoff values are mostly for “help-seeking” population; recommendations of the total symptoms score's threshold for the general population are not available. We chose a threshold of ≥3, which has shown a sensitivity of 100% and a specificity of 52% according to Ising et al. (2012). No defined cutoff values are available for the HAPSY-Q. For the M.I.N.I. psychosis module, the established rating score was used, which scores overt psychosis either “lifetime” or “present” (Sheehan et al., 1998).

### Statistical analysis

**FIG. 2.**
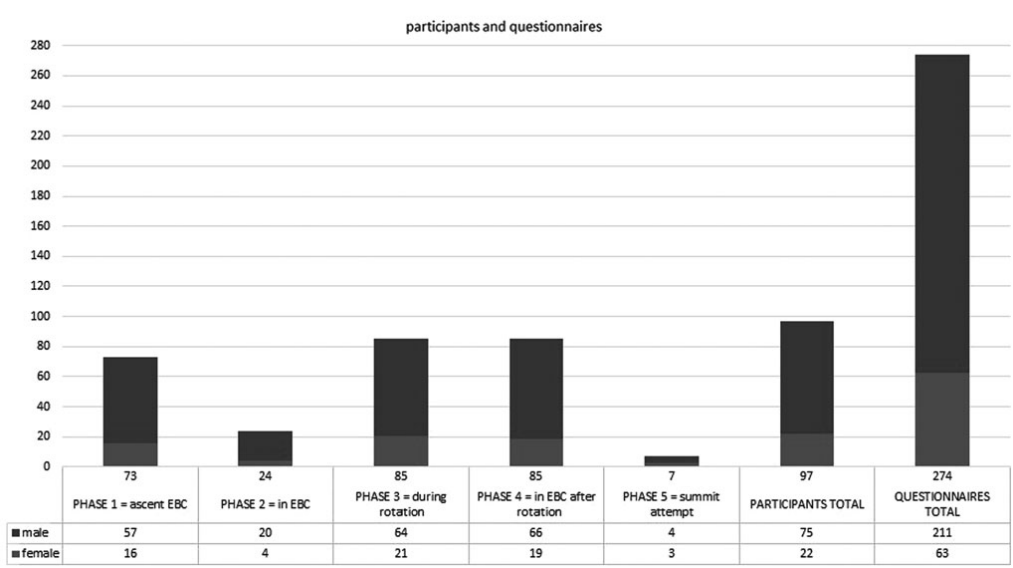
Participants and questionnaire numbers divided by phases as well as the total number of participants and questionnaires analyzed in the study. The male/female ratio is symbolized by *dark* and *light shades*.

To allow for statistical analysis, the completed questionnaires had to be divided into different phases depending on the altitude evaluated. The following phases were used: phase 1—ascent to EBC, phase 2—in EBC before reaching higher altitudes (duration of 7.7 ± 6.8 days; mean ± standard deviation), phase 3—period of rotations ( = the period of HA acclimatization climbs; altitude range: 5,300–7,400 m), phase 4—in EBC following rotations (duration of 19.7 ± 6.2 days; mean ± standard deviation), and phase 5—summit attempt (Fig. 2).

We analyzed two sets of factors: the phase-independent factors, which were stable between the different climbing phases with no change during the expedition or even lifetime (such as sex or lifetime history of psychosis, Table 1), and the phase-dependent factors, which change from phase to phase during the expedition (such as, e.g., AMS symptoms or cough, Table 2).

**Table 1.** Sociodemographics, Stable Lifestyle Factors, and Stable Factors Related to the Climb

Stable factors	
Total participants	100% (97/97)
Sex	
Female	22.7% (22/97)
Occupation	
Businessmen	15.5% (15/97)
Mountain guide	14.4% (14/97)
Doctor/paramedic	12.4% (12/97)
Military/army/police	9.3% (9/97)
Ethnicity	
Caucasian	56.3% (54/96)
Asian	39.6% (38/96)
Hispanic	2.1% (2/96)
African	2.1% (2/96)
HA natives (>2,500 m)	5.3% (5/94)
Lifestyle factors	
Regular smoker before expedition	
Yes	7.2% (7/97)
No	79.4% (77/97)
Ex-smoker	13.4% (13/97)
Regular alcohol before expedition	37.1% (36/97)
Recreational drug use before expedition^a^	50% (39/78)
Pre-existing health conditions^b^	21.6% (21/97)
Acclimatization	
Prior altitude exposure	
Yes, in the last 3 months	44.3% (43/97)
Yes, not in the last 3 months	23.7% (23/97)
Acclimatization peak^c^	
Yes	34.1% (29/85)
Setting	
Starting point of active trekking	
2,860 m (Lukla)	88.7% (86/97)
3,440 m (Namche Bazar)	11.3% (11/97)
Targeted peak	
Mt. Everest	77.2% (61/79)
Lhotse	10.1% (8/79)
Mt. Everest+Lhotse	6.3% (5/79)
Mt. Everest+Lhotse+Makalu	1.3% (1/79)
None	5.1% (4/79)
History of HA diseases	
Prior AMS	26.2% (22/84)
Prior HACE	2.4% (2/82)
Prior HAPE	3.7% (3/82)
M.I.N.I.	
M.I.N.I. positive lifetime—no organic cause	3.5% (3/85)
M.I.N.I. positive lifetime—all cases	9.4% (8/85)
Single psychotic symptoms in lifetime caused by^d^	29.4% (25/85)
Altitude	9.5% (8/84)
Drugs	7.1% (6/84)
Meditation/yoga	2.4% (2/84)
Exhaustion	7.1% (6/84)
Others	7.1% (6/84)

Data are given as % as well as absolute numbers. For the absolute numbers, the number of affirmative scorings is given together with the total count of answers available for this question.

^a^ Includes in decreasing frequencies cannabis, hallucinogenics (mushrooms and LSD), stimulants (cocaine and XTC), and other (opioids, benzodiazepines, and not better specified as “pills”).

^b^ Including respiratory tract (5/97): asthma and recurring sinusitis; neurological/psychiatric (5/97): epilepsy, mental health conditions, and syringomyelia; gastrointestinal tract/urinary tract (4/97): Meckel-diverticulum, achalasia, biliary colic, and problems in urinary tract (not better specified); immunological system/cancer/hematological (4/97): hemochromatosis, breast cancer, and low immunity; musculoskeletal system (3/97): lumbago, disc bulge, rhabdomyolysis, and knee-related conditions.

^c^ Mera Peak 6,461 m, Island Peak 6,189 m, Lobuche Peak 6,119 m, and Kala Patthar 5,643 m.

^d^ Includes eight individuals with a positive lifetime M.I.N.I. scoring and additional individuals who reported single psychotic symptoms in their past medical history, but did not fulfill M.I.N.I. scoring criteria (i.e., only reported hallucinations, but no other psychotic symptoms).

AMS, acute mountain sickness; HA, high altitude; HACE, high altitude cerebral edema; HAPE, high altitude pulmonary edema; LSD, lysergic acid diethylamide (hallucinogenic drug); M.I.N.I., Mini International Neuropsychiatric Interview; XTC, 3,4-methylenedioxymethamphetamine (MDMA; psychoactive drug).

**Table 2.** Phase-Dependent Lifestyle and Medical Factors, High Altitude Somatic Conditions As Well As Accidents

	Phase-dependent factors				
	Phase 1	Phase 2	Phase 3	Phase 4	Phase 5
Total questionnaires	26.6% (73/274)	8.8% (24/274)	31.0% (85/274)	31.0% (85/274)	2.6% (7/274)
Caffeine	52.8% (38/72)	73.9% (17/23)	53.2% (42/79)	60.0% (51/85)	80.0% (4/5)
Black tea	76.7% (56/73)	58.3% (14/24)	74.7% (59/79)	80.0% (68/85)	80.0% (4/5)
Hunger/starvation	11.0% (8/73)	8.3% (2/24)	11.4% (9/79)	4.7% (4/85)	20.0% (1/5)
Dehydration	9.6% (7/73)	16.7% (4/24)	13.9% (11/79)	1.2% (1/85)	50.0% (2/4)
Regular smoker since start of expedition	4.1% (3/73)	4.2% (1/24)	3.5% (3/85)	3.5% (3/85)	0% (0/7)
Regular alcohol since start of expedition	25.0% (18/72)	25.0% (6/24)	21.7% (18/83)	21.4% (18/84)	28.6% (2/7)
Recreational drug use since start of expedition	1.4% (1/71)	0% (0/23)	1.2% (1/83)	1.2% (1/84)	0% (0/7)
Illness since start of expedition^a^	52.1% (38/73)	45.8% (11/24)	57.6% (49/85)	55.3% (47/85)	71.4% (5/7)
Medication					
Diamox	16.7% (12/72)	8.3% (2/24)	30.6% (26/85)	15.3% (13/85)	14.3% (1/7)
Analgesics/NSAID	18.1% (13/72)	12.5% (3/24)	25.9% (22/85)	5.9% (5/85)	28.6% (2/7)
Oxygen	1.4% (1/73)	4.2% (1/24)	6.5% (5/77)	3.6% (3/83)	100% (6/6)
Antibiotics	6.9% (5/72)	4.2% (1/24)	4.7% (4/85)	9.4% (8/85)	0% (0/7)
Anticholinergics	0% (0/72)	0% (0/24)	7.1% (6/85)	4.7% (4/85)	28.6% (2/7)
Antihistamines	2.8% (2/72)	0% (0/24)	0% (0/85)	1.2% (1/85)	0% (0/7)
Psychotropic drugs^b^	2.8% (2/72)	0% (0/24)	1.2% (1/85)	1.2% (1/85)	0% (0/7)
Experienced symptoms					
Cough	23.3% (17/73)	25.0% (6/24)	55.7% (44/79)	48.2% (41/85)	42.9% (3/7)
Snow blindness	0% (0/73)	0% (0/24)	1.3% (1/80)	0% (0/85)	0% (0/7)
Fever	6.1% (4/66)	0% (0/20)	9.4% (6/64)	0% (0/40)	0% (0/3)
Headache	37.0% (27/73)	16.7% (4/24)	35.3% (30/85)	1.2% (1/85)	28.6% (2/7)
Lake Louis Self-Rating Score					
Diagnosis of AMS	6.8% (5/73)	0% (0/24)	12.9% (11/85)	1.2% (1/85)	16.7% (1/6)
HAPE Scoring-Algorithm					
Diagnosis of HAPE	3.1% (2/65)	0% (0/23)	3.5% (2/57)	0% (0/70)	0% (0/1)
HACE Scoring-Algorithm					
Diagnosis HACE	0% (0/71)	0% (0/23)	0% (0/70)	0% (0/73)	0% (0/1)
Neurological assessment					
Normal alertness	100% (72/72)	100% (23/23)	100% (70/70)	100% (74/74)	100% (2/2)
Disorientation	5.6% (4/72)	0% (0/23)	0% (0/70)	0% (0/74)	0% (0/3)
Ataxia	0% (0/71)	0% (0/23)	0% (0/70)	0% (0/74)	0% (0/3)
Accidents					
Accident/near accident	1.4% (1/73)	8.3% (2/24)	6% (5/84)	0% (0/84)	0% (0/7)

Data are given as % as well as absolute numbers. For the absolute numbers, the number of affirmative scorings is given together with the total number of answers available for this question.

^a^ Including respiratory tract symptoms, gastrointestinal symptoms, urinary tract infection, eye infection, and retinal hemorrhage, as well as unspecific symptoms such as loss of energy.

^b^ Including Sertralin 100 mg/day, not better specified SSRI 150 mg, not better specified benzodiazepine.

AMS, acute mountain sickness; HACE, high altitude cerebral edema; HAPE, high altitude pulmonary edema; SSRI, selective serotonin reuptake inhibitor.

Data are presented as mean, minimum, maximum, standard deviation, and confidence intervals. To account for missing data, the number of individuals who completed a given item is always indicated.

Nonparametric correlation analysis was performed using Spearman's rank correlation. Cronbach's alpha was used for internal consistency to test for reliability of the test (Tavakol and Dennick, 2011). Odds ratios were calculated for possible risk factors of single symptoms of psychosis. Calculating the odds ratio requires binary variables and therefore only factors that could be presented in a binary way were included (Simon, 2001). All data were analyzed using IBM SPSS Statistics Version 25 (IBM Cooperation) and graphically presented by using Microsoft Excel 2019.

## Results

### Participants

Ninety-nine climbers or accompanying staff with sufficient command of the English language, who gave informed consent, were included in the study. Since climbers participated in multiple phases during the climb, this resulted in the collection of 279 questionnaires. Two participants were excluded from the study (one individual because of implausible values and the second because of pre-existing health conditions, a brain hemorrhage caused by a cavernoma, interfering with the diagnosis of HA psychosis). Data from 97 participants with a total of 274 questionnaires (the latter being referred to as “cases” in the following text) were analyzed. Thirty-six participants were assessed twice, 43 participants thrice, 17 participants completed four questionnaires, and 1 participant completed five questionnaires. Sociodemographic data as well as factors related to the climb can be found in Table 1.

### Stable factors relevant for the HA climb

Analysis of the stable factors showed the mean age of participants as 39 ± 10 years (mean ± standard deviation; four missing data) and 22.7% were female. The majority of climbers was of Caucasian or Asian ethnicity (Table 1). Physical training during the 2 months before the expedition was 16.6 ± 6.5 hours/week (mean ± standard deviation; 41 missing data).

The M.I.N.I. psychosis module identified eight individuals with a positive scoring for “lifetime,” three of these with no organic causes and five with underlying organic causes (or similar provocative situations): two positive scoring were attributed to drug-induced psychotic symptoms, one was related to meditation, one to exhaustion, and in one participant, a provoking situation was assumed during the interview, but the exact nature of the provoking agent was not well documented. Some individuals reported single psychotic symptoms without scoring positive for a M.I.N.I. psychosis diagnosis; these are also reported in Table 1.

### Phase-dependent factors and somatic symptoms relevant for the HA climb

The phase-dependent factors are shown in Table 2, divided by phases. Diagnosis of AMS was low, between 0% and 16.7% depending on the altitude: 5 participants (5/73 = 6.8%) in phase 1, 0 participants (0/24) in phase 2, 11 participants (11/85 = 12.9%) in phase 3, 1 participant (1/85 = 1.2%) in phase 4, and 1 participant (1/6 = 16.7%) in phase 5. Over all phases, 18 individuals were diagnosed with AMS and 4 with HAPE. Neurological assessment showed no case of HACE, but four participants with disorientation in phase 1 (during ascent to EBC). Further data on the physical examination can be found in the Supplementary Table S1. All participants were on organized expeditions during the whole climbing period, and no participant was climbing alone (Supplementary Table S1). During phases 3–5, one or two individuals each were sleeping alone in a tent (Supplementary Table S1). Analysis of the phase-dependent factors showed eight cases with accidents or near accidents occurring in six individuals (two individuals with two cases each): two accidents/near accident cases were attributed to natural disaster and six to human error.

### HA-psychiatric symptoms

Table 3 shows the results of the psychometric scorings and the M.I.N.I. The maximum score endorsed in the HAPSY-Q was 2 (the HAPS-Q consists of 11 items), while it was 9 on the PQ-16 (the PQ-16 consists of 16 items). Two hundred seventy-four HAPSY-Q questionnaires were answered: 261 of them with no endorsed item, 9 cases with one endorsed item, and 4 cases with two endorsed items (Table 3). These positive scorings referred to 10 different participants [10/97 (10.3%)]. Two hundred thirty-five PQ-16 questionnaires were completed, with 203 cases with no positive answer, 18 cases with one endorsed item, 7 cases with two endorsed items, 2 cases with three endorsed items, 2 cases with four endorsed items, and 1 case each with five, seven, and nine endorsed items. At least one endorsed item was found in 32 cases attributed to 18 participants [18/87 (20.7%)]. The seven cases exceeding our chosen cutoff threshold of ≥3 were attributed to four participants.

**Table 3.** Analysis of Psychometric Tests

	Phase 1	Phase 2	Phase 3	Phase 4	Phase 5
CAM					
Diagnosis of delirium	(0/70)	(0/23)	(0/70)	1.4% (1/74)	(0/2)
HAPSY-Q					
0 pts	97.3% (71/73)	100% (24/24)	92.9% (79/85)	94.1% (80/85)	100% (7/7)
1 pt	1.4% (1/73)	(0/24)	4.7% (4/85)	4.7% (4/85)	(0/7)
2 pts	1.4% (1/73)	(0/24)	2.4% (2/85)	1.2% (1/85)	(0/7)
≥1 pts	2.7% (2/73)	(0/24)	7.1% (6/85)	5.9% (5/85)	(0/7)
PQ-16					
0 pts	88.9% (64/72)	87.0% (20/23)	85.7% (54/63)	85.1% (63/74)	66.7% (2/3)
1 pt	4.2% (3/72)	(0/23)	9.5% (6/63)	10.8% (8/74)	33.3% (1/3)
2 pts	4.2% (3/72)	8.7% (2/23)	1.6% (1/63)	1.4% (1/74)	(0/3)
3 pts	(0/72)	(0/23)	1.6% (1/63)	1.4% (1/74)	(0/3)
4 pts	1.4% (1/72)	(0/23)	(0/63)	1.4% (1/74)	(0/3)
5 pts	(0/72)	(0/23)	1.6% (1/63)	(0/74)	(0/3)
6 pts	(0/72)	(0/23)	(0/63)	(0/74)	(0/3)
7 pts	1.4% (1/72)	(0/23)	(0/63)	(0/74)	(0/3)
8 pts	(0/72)	(0/23)	(0/63)	(0/74)	(0/3)
9 pts	(0/72)	4.3% (1/23)	(0/63)	(0/74)	(0/3)
≥1 pts	11.1% (8/72)	13.0% (3/23)	14.3% (9/63)	14.9% (11/74)	33.3% (1/3)
≥3 pts	2.8% (2/72)	4.3% (1/23)	3.2% (2/63)	2.7% (2/74)	(0/3)
HAPSY-Q and PQ-16 positive scoring simultaneously					
HAPSY-Q and PQ-16 ≥ 1 pt	2.7% (2/73)	(0/24)	3.2% (2/63)	1.4% (1/74)	(0/3)
HAPSY-Q ≥ 1 and PQ-16 ≥ 3 pts	1.4% (1/73)	(0/24)	3.2% (2/63)	1.4% (1/74)	(0/3)
M.I.N.I. (psychosis module)					
M.I.N.I. positive currently	(0/72)	(0/23)	1.4% (1/70)	(0/74)	No valid cases

Data are given as % as well as absolute numbers. For the absolute numbers, the number of affirmative scorings is given together with the total number of answers available for this question.

CAM, Confusion Assessment Method for the diagnosis of delirium; HAPSY-Q, High Altitude Psychosis Questionnaire; pts, points; PQ-16, Prodromal Questionnaire 16; M.I.N.I., Mini International Neuropsychiatric Interview.

One individual with a positive M.I.N.I. scoring for the psychosis module “currently” was identified in phase 3. This case occurred in a participant, who, in addition to the positive M.I.N.I. scoring during rotation, also showed a positive M.I.N.I. psychosis module result during lifetime. Moreover, two endorsed items in HAPSY-Q and five endorsed items in PQ-16 were recorded. Fever and chest tightness/congestion were reported for this participant during phase 3. The person had a history of prior AMS, but no AMS during the rotation (phase 3), where the positive M.I.N.I. psychosis module scoring was observed. No diagnosis of delirium was made during phase 3, but in a later phase (phase 4).

### Comparison of the HAPSY-Q, PQ-16, and M.I.N.I. scores

Cronbach's alpha for the HAPSY-Q showed alpha values between 0.28 and 0.59 depending on the analyzed phase (Table 4), the values for the PQ-16 using phase per phase analysis resulted in values between 0.60 and 0.96 (Table 4). The overall data showed a Cronbach's alpha for the HAPSY-Q of 0.36 and for the PQ-16 of 0.80. Using correlation analysis, two of the five phases (phase 1 and phase 3) showed a significant correlation of the HAPSY-Q and the PQ-16 scorings, one phase (phase 4) showed no correlation, and for two phases (phase 2 and phase 5), there was no valid value. A significant correlation between the HAPSY-Q and the PQ-16 using the overall data was found (Table 4).

**Table 4.** Cronbach's Alpha and Correlation Analyses for High Altitude Psychosis Questionnaire and Prodromal Questionnaire 16

	Cronbach's alpha HAPSY-Q		Cronbach's alpha PQ-16		Correlation HAPSY-Q vs. PQ-16		
	Alpha	N_alpha_	Alpha	N_alpha_	Correlation coefficient (*r*)	p value two tailed	N
Phase 1	0.59	3	0.83	11	0.48	<0.001	72
Phase 2	Not valid	/	0.97	10	Not valid	Not valid	23
Phase 3	0.38	5	0.74	7	0.31	0.02	63
Phase 4	0.28	5	0.60	9	0.14	0.24	74
Phase 5	Not valid	/	Not valid	/	Not valid	/	3
Overall data	0.36	6	0.80	13	0.27	<0.001	235

Not valid: indicates that no positive scoring was available in this phase for scoring.

*N*, number of cases included in the correlation analysis; *N*_alpha_, number of items included in the calculation for the Cronbach's alpha analysis.

### Symptoms of psychosis: protective and risk factors and the association with accidents

Factors related to the individual or the climb were assessed for their potential to convey risk factors of single symptoms of psychosis at HA using odds ratio analysis. Potentially protective factors such as caffeine use were identified (Table 5 in dark gray), while other factors such as medication intake or diagnosis of AMS were associated with an increased risk for perceptual instability (Table 5 in light gray). Positive endorsed items on the HAPSY-Q were identified as risk factor for accidents and near accidents. Some factors showed inconclusive results.

**Table 5.** Odds Ratio for Single Symptoms of Psychosis as Measured by High Altitude Psychosis Questionnaire or Prodromal Questionnaire 16

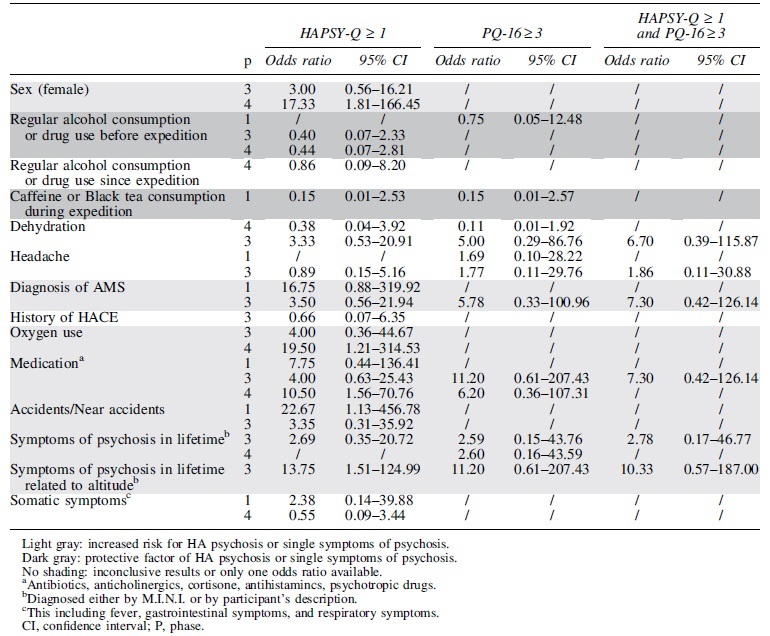

## Discussion

The main findings of this field study performed at EBC were as follows: (1) only one individual (1/97) fulfilled the diagnostic criteria for psychosis during HA exposure according to M.I.N.I. diagnostic interview; (2) at least one endorsed item on the HAPSY-Q and the PQ-16, indicating the presence of symptoms of psychosis (in the absence of a psychotic disorders), was identified in 10/97 (10.3%) and 18/86 (20.9%) participants, respectively; (3) scorings of the HAPSY-Q and the PQ-16 correlated with each other; (4) internal consistency for the HAPSY-Q was poor, while it was acceptable for the PQ-16; (5) the possible risk factors for single symptoms of psychosis were identified; and (6) positive endorsed items on the HAPSY-Q was found to be a risk factor for accidents and near accidents.

The observed values for the PQ-16 and the HAPSY-Q were low overall, that is, there were not many questionnaires with positively endorsed items. For the PQ-16, different cutoff values have been reported in the literature to identify individuals of UHR of psychosis. These cutoff values were all defined for “help-seeking” populations (Ising et al., 2012; McDonald et al., 2019), while cutoff values for the general population could, in theory, be even higher (Savill et al., 2018). We suggest that the observed scorings in most cases do not identify cases of prodromal symptoms of psychosis, but they might identify single symptoms of psychosis (e.g., only visual hallucinations) pointing toward different levels of perceptual instability (Horga and Abi-Dargham, 2019). No cutoff score could be defined for the HAPSY-Q in this study since there were not enough cases with positive scorings. Single psychotic symptoms have been found in the community population at sea level without a diagnosable disorder at various frequencies (Nuevo et al., 2012), and these symptoms can impact an individual`s capability to function within daily life, even if symptoms do not reach the clinical threshold for a disorder. In addition, single symptoms of psychosis have been shown to be related to impaired physical health as well as accidents such as road traffic accidents at sea level (Moreno et al., 2013).

The internal consistency of the HAPSY-Q was poor, and the PQ-16 showed better values. This is surprising since both questionnaires showed a positive correlation for most phases analyzed. One fact contributing the insufficient internal consistency of HAPSY-Q might be that there were overall fewer questions to be answered compared to the PQ-16 and that there were fewer cases and individuals who endorsed items on the HAPSY-Q compared to the PQ-16. Possibly, the questionnaires could be better evaluated in individuals with a psychotic disorder at sea level. However, the problem would be that specific symptoms of isolated HA psychosis, such as the third man phenomenon (i.e., hallucinations pointing toward the presence of an additional person), would not be captured adequately. The HAPSY-Q has been designed specifically to detect psychotic symptoms frequently observed at HA by HA climbers and psychiatrists (Hufner et al., 2019).

The one positive M.I.N.I. psychosis module scoring, which we observed in one participant, probably has to be attributed to delirium due to a respiratory infection. This individual showed fever and chest tightness during the phase of the positive M.I.N.I. psychosis module scoring. The formal diagnosis of delirium using the CAM was only made in the subsequent stage, but nevertheless, this seems the most likely diagnosis.

With only one confirmed case, the prevalence of psychosis in our sample was very low. This might be due to the fact that all participants were on organized expeditions. No participant slept alone in a tent while in EBC, and only two participants slept alone in a tent during rotations (Supplementary Table S1). Therefore, there was no social deprivation, lower risk, and probably a sense of security conveyed by the organizers. The fact that social deprivation is not present during the peak climbing season on Mt. Everest was illustrated in 2019, the year the study was performed, by the photographs in the media showing a long queue to the top (e.g., on a report on https://www.nationalgeographic.de) (Wilkinson, 2020). This might be protective against HA psychosis since sensory or social isolation has been shown to be a risk factor for psychosis outside the HA setting (Daniel et al., 2014; O'Donoghue et al., 2016). For climbers performing solo or alpine-style ascents in remote areas, the setting and circumstances are completely different and would need to be assessed separately, which could lead to different results (Carbone et al., 2020).

It should be pointed out that the practical experiences in administering the HAPSY-Q as well as the PQ-16 were very positive. Participants reported no problem with their completion and completion was fast and easy.

As in previous research, we found an association of single symptoms or full-blown psychosis with accidents and near accidents (Hufner et al., 2018). This is concerning and should alert climbers and expedition leaders alike since it shows that not only full blown psychosis but also more subtle and single symptoms can lead to detrimental situations during HA activities, making early and reliable recognition of such symptoms very important.

Diagnosis of delirium (one case) was quite low in this study, compared to previous literature (Ryn, 1988), possibly due to the fact that ascent was gradual with good acclimatization times and that the number of individuals who were questioned following the summit attempt was low. When a diagnosis of delirium is made, it is always essential to try and treat the underlying pathology, which was probably of infectious origin in this case. However, at HA, it is always important to also consider HACE as a cause of delirium due to the consecutive implications for treatment.

It is difficult to compare prevalence AMS numbers between studies since the most significant risk factor for AMS is the rate of ascent and following acclimatization symptoms of AMS usually improve or subside (Gallagher and Hackett, 2021). In our study, AMS rates were quite low, but were consistent with the climbing profiles of the climbers, that is, higher rates were found for phases 1, 3, and 5, in which the climbers ascended to higher altitudes, while lower rates were found for phases 2 and 4, in which climbers were in EBC before or following rotations to higher altitudes. Sampling bias (see Limitations section) might also play a role in these numbers.

### Limitations

In this study, it was not possible to identify the psychometric properties of the HAPSY-Q due to the low number of endorsed items. This low number of affirmative scorings might, in part, be due to the fact that in this study, we probably examined a sample with a relatively low prevalence of severe mental health conditions since individuals with an active, untreated psychotic disorder potentially face many barriers in climbing (there is no official account of an individual living with schizophrenia having climbed Mount Everest to date). Unfortunately, in this field study, it was not possible to include an interview of every participant during every phase of the climb and only individuals in a physical and mental state of completing a questionnaire and physical examination were included, which probably lead to a sampling bias. Especially after the summit attempt, the stay in the EBC was very short. When interpreting the odds ratio analysis of risk and protective factors of psychotic symptoms, it is important to keep in mind the at times large confidence intervals.

## Conclusions

The diagnosis of HA psychosis is rare in a population of climbers who participate in organized commercial expeditions at Mount Everest. Nevertheless, it is a finding of significant practical relevance that single psychotic symptoms occurred in a significant proportion of climbers, and single endorsed items on the HAPSY-Q conveyed a possible risk factor for near accidents or accidents. In the setting of organized commercial expeditions, it was not possible to evaluate the psychometric properties of the HAPSY-Q and compare it to the PQ-16. It might be advisable to re-evaluate the questionnaire on climbers during solo or alpine-style ascents in remote high mountain areas, who are exposed to higher levels of altitude, sensory deprivation, and physical and mental stress over a longer period time.

## Supplementary Material

Supplemental data
