# Supplementary material for: Assessment of Psychotic Symptoms in Individuals Exposed to Very High or Extreme Altitude: A Field Study
Source: High Alt Med Biol. 2021 Dec 13;22(4):369–78. doi: 10.1089/ham.2020.0210 (PMC8742264; doi:10.1089/ham.2020.0210)
Supplement: Supplemental data [file Supp_Table1.docx]

**Supplemental material**

| **Phase-dependent FACTORS** | | | | | | | |
| --- | --- | --- | --- | --- | --- | --- | --- |
|  |  | Phase 1 | Phase 2 | Phase 3 | Phase 4 | Phase 5 | |
| Special diet | vegetarian/vegan | 11.1% (8/72) | 16.7% (4/23) | 8.5% (7/82) | 7.1% (6/85) | (0/4) | |
|  | other | 13.9% (10/72) | 8.7% (2/23) | 12.2% (10/82) | 15.3% (13/85) | (0/4) | |
| Nutritional supplement* |  | 37.0% (27/73) | 50.0% (12/24) | 31.3% (25/80) | 35.7 (30/84) | 50.0% (2/4) | |
| Fluid consumption per day [liters] | mean +/- SD | 3.09 +/- 0.95 | 2.9 +/- 0.74 | 3.03 +/- 0.97 | 3.27 +/- 0.83 | 2.90 +/- 1.25 | |
|  | abs. number | (71/73) | (24/24) | (77/85) | (82/85) | (5/7) | |
| Solo ascent | unsupported expedition | (0/73) | (0/24) | (0/84) | (0/84) | (0/7) | |
|  | climbing without partner | (0/73) | (0/24) | (0/84) | (0/84) | (0/7) | |
|  | sleeping in tent alone | (0/73) | (0/24) | 2.4% (2/84) | 1.2% (1/84) | 14.3% (1/7) | |
| Physical evaluation | |  |  |  |  |  | |
| Heart-rate in beats/min | mean +/-SD | 86.36 +/-22.69 | 90.35 +/-14.67 | 91.09 +/-20.21 | 84.85 +/-12.73 | / | |
|  | abs. number | (25/73) | (23/24) | (22/85) | (73/85) | (0/7) | |
| Systolic blood pressure  Diastolic blood pressure | mean +/-SD | 100.00 +/-0 | 128.32 +/-14.23 | 120.00 +/-0 | 127.10 +/-19.34 | / | |
|  | abs. number | (1/73) | (22/24) | (1/85) | (73/85) | (0/7) | |
|  | mean +/-SD | 101.00 +/-41.01 | 85.86 +/-10.70 | 80.00 +/-0 | 88.00 +/-11.14 | / | |
|  | abs. number | (2/73) | (22/24) | (1/85) | (72/85) | (0/7) | |
| Respiratory rate | mean +/-SD | 12.00 +/-0 | 13.04 +/-1.80 | / | 15.19 +/-3.15 | / | |
|  | abs. number | (1/73) | (23/24) | (0/85) | (54/85) | (0/7) | |
| Oxygen saturation (SpO2%) | mean +/-SD | 86.00 +/-0 | 79.20 +/-3.71 | / | 82.54 +/-3.92 | / | |
|  | abs. number | (1/73) | (20/24) | (0/85) | (71/85) | (0/7) | |
| Accidents | |  |  |  |  |  | |
| Accident since start of expedition | | 1.4%  (1/73) | 4.2%  (1/24) | 2.4% (2/84) | (0/84) | | (0/7) |
| Accident cause | human error | 1.4% (1/73) | 4.2% (1/24) | 2.4% (2/84) | (0/84) | | (0/7) |
| Near-accident since start of expedition | | (0/73) | 4.2% (1/24) | 3.6% (3/84) | (0/84) | | (0/7) |
| Near-accident cause | natural disaster | (0/73) | 4.2% (1/24) | 1.2% (1/84) | (0/84) | | (0/7) |
|  | human error | (0/73) | (0/24) | 2.4% (2/84) | (0/84) | | (0/7) |

Additional phase-dependent factors not reported in the main analysis. For metric values an extra line was inserted with absolute numbers indicating the individuals who had undergone this assessment in relation to all individuals.

*vitamins, iron, proteins, potassium, magnesium-either alone or in combination
